# Pharmacological Characterization of Dezocine, a Potent Analgesic Acting as a κ Partial Agonist and μ Partial Agonist

**DOI:** 10.1038/s41598-018-32568-y

**Published:** 2018-09-20

**Authors:** Yu-Hua Wang, Jing-Rui Chai, Xue-Jun Xu, Ru-Feng Ye, Gui-Ying Zan, George Yun-Kun Liu, Jian-Dong Long, Yan Ma, Xiang Huang, Zhi-Chao Xiao, Hu Dong, Yu-Jun Wang

**Affiliations:** 1 School of Pharmacy, Nanjing University of Chinese Medicine, Nanjing, 210046 China; 2 Key Laboratory of Receptor Research, Shanghai Institute of Materia Medica and Collaborative Innovation Center for Brain Science, Chinese Academy of Science, Shanghai, 201203 China; 3 Dublin Coffman High School, Dublin, OH 43017 USA; 4 School of Life Sciences, Shanghai University, Shanghai, 200444 China

## Abstract

Dezocine is becoming dominated in China market for relieving moderate to severe pain. It is believed that Dezocine’s clinical efficacy and little chance to provoke adverse events during the therapeutic process are mainly attributed to its partial agonist activity at the μ opioid receptor. In the present work, we comprehensively studied the pharmacological characterization of Dezocine and identified that the analgesic effect of Dezocine was a result of action at both the κ and μ opioid receptors. We firstly found that Dezocine displayed preferential binding to μ opioid receptor over κ and δ opioid receptors. Dezocine, on its own, weakly stimulated G protein activation in cells expressing κ and μ receptors, but in the presence of full κ agonist U50,488 H and μ agonist DAMGO, Dezocine inhibited U50,488H- and DAMGO-mediated G protein activation, indicating that Dezocine was a κ partial agonist and μ partial agonist. Then the *in intro* results were verified by *in vivo* studies in mice. We observed that Dezocine-produced antinociception was significantly inhibited by κ antagonist nor-BNI and μ antagonist β-FNA pretreatment, indicating that Dezocine-mediated antinociception was via both the κ and μ opioid receptors. When co-administrating of Dezocine with U50,488 H or morphine, Dezocine was capable of inhibiting U50,488H- or morphine-induced antinociception. Finally, κ receptor activation-associated side effect sedation was investigated. We found that Dezocine displayed limited sedative effect with a ceiling effecting at a moderate dose. Thus, our work led to a better understanding of the analgesic mechanism of action of Dezocine *in vivo*.

## Introduction

Morphine, fentanyl and all related opioid drugs used for relief of moderate and severe pain in clinic are agonists at μ opioid receptors^1,2^. These prototypical μ agonists produced potent analgesia, and also mediated adverse effects including liability to addiction, respiratory depression, constipation and urinary retention, by action at μ opioid receptors^3^. An approach to limiting the undesirable side effects, but still have therapeutic potential as potent analgesics is to develop compounds that possess mixed agonist-antagonist or partial agonist activities at different opioid receptors, since activation of the different opioid receptors may have synergistic effects or may produce fewer side effects than acting at a single target^1,4–7^. The well-known mixed agonist/antagonist analgesics include pentazocine, butorphanol, nalbuphine, buprenorphine and Dezocine^1,7^, among which, Dezocine, a synthesized bridged aminotetralin^4,8–10^, currently is a prescribed painkiller in China for moderate to severe pain treatment. In 2016, sales of Dezocine reached more than $630 million nationally, with occupying over 45% of the nation’s opioid analgesics market.

Early studies demonstrated that Dezocine produced more potent or equipotent analgesia than morphine in rodents, primates and humans, due to its μ agonist (morphine-like) activity^10^. The evidence came from both *in vitro* functional studies^11^ and *in vivo* drug antinociceptive^12^ and discriminative stimulus studies^13^. Meanwhile, Dezocine exhibited certain μ antagonist activity as showing by the fact that Dezocine produced antagonism on morphine-induced loss of righting reflex, although Dezocine did not significantly precipitate withdrawal jumping syndrome in chronically morphine-treatment mice^10^. Compared with opioid analgesics acting as μ full agonists such as morphine, Dezocine has a safe and tolerable side-effects profile, including relatively low analgesic tolerance^14^, lack of physical dependence capacity^10,15^ and limited respiratory depression^10,15,16^. According to these observations, Dezocine seems to perform as a μ partial agonist, which can explain its unique profile. Indeed, Liu with his colleagues recently characterized molecular targets for Dezocine and provided reliable evidence supporting that Dezocine acted as a μ partial agonist^17^. In addition to μ opioid receptors, Dezocine also interacted with κ opioid receptors, however, the profile of Dezocine at κ receptors remained controversial. Due to its structurally related to Pentazocine, a κ agonist and μ partial agonist, Dezocine was initially identified as a κ agonist^4^. Subsequent studies from Gharagozlou *et al*. (2006) and Liu *et al*. (2014) by using *in vitro* functional assay demonstrated that Dezocine acted as a κ antagonist^17,18^. At present, *in vivo* data regarding to its activity at κ opioid receptor is lacking. Whether Dezocine-induced analgesia was partially a result of action at κ receptor and κ receptor-associated side effects were unknown.

Thus, the present work was undertaken to comprehensively address the pharmacological properties of Dezocine. We found that Dezocine displayed preferential binding to μ opioid receptor over κ and δ opioid receptors, and acted as a κ partial agonist and μ partial agonist, evaluated by functional [^35^S]GTPγS binding assay. *In vivo* studies, Dezocine produced more potent antinociception than morphine, which was via activation of both the κ and μ opioid receptors. Dezocine was capable of inhibiting κ agonist- and μ agonist-induced antinociception. In addition, Dezocine displayed limited sedative effects with a ceiling effecting at a moderate dose.

## Results

### Affinity, selectivity and efficacy of Dezocine

**Figure 1:**
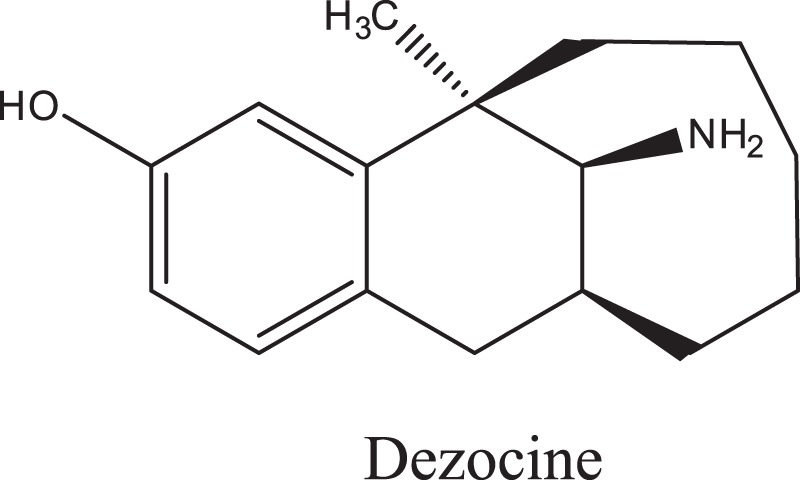
The chemical structure of Dezocine.

**Figure 2:**
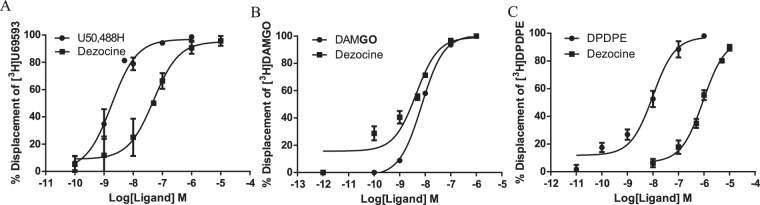
Displacement of [^3^H]ligands binding to κ, μ and δ opioid receptors by Dezocine and reference compounds. Each data point represents the mean ± SEM of at least three independent experiments conducted in triplicate. *Ki* values are shown in Table 1.

Figure 1 depicts the structure of Dezocine. Competitive inhibition of [^3^H]ligands ([^3^H]U69593, [^3^H]DAMGO and [^3^H]DPDPE) binding to opioid receptors (κ, μ and δ) by Dezocine was conducted to examine the binding affinities of Dezocine. As shown in Fig. 2A–C and Table 1, Dezocine had *Ki* values of 1.46 nM, 22.01 nM and 398.6 nM for μ, κ and δ opioid receptors, respectively. The data indicated that Dezocine displayed preferential binding to μ opioid receptors over κ and δ opioid receptors.

**Table 1:** Affinity values (*Ki*) for the binding to human κ-, rat μ, or rat δ-opioid receptors.

Compounds	µ binding affinity *K*_*i*_ (nM)	δ binding affinity *K*_*i*_ (nM)	κ binding affinity K_i_ (nM)
Dezocine	1.46 ± 0.10	398.6 ± 43.25	22.01 ± 1.52
DAMGO	2.30 ± 0.02	—	—
DPDPE	—	4.36 ± 0.69	—
U50,488 H,	—	—	0.91 ± 0.20

Membranes were incubated with varying concentrations of ligands in the presence of 2.0 nM [^3^H]DAMGO, 1.1 nM [^3^H]DPDPE and 1.5 nM [^3^H]U69593. Data are expressed as the mean ± SEM for at least three independent experiments performed in triplicate.

**Figure 3:**
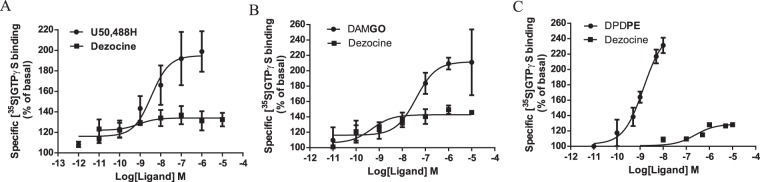
Stimulation of [^35^S]GTPγS to κ, μ and δ opioid receptors by Dezocine and reference compounds. Each data point represents the mean ± SEM of at least three independent experiments conducted in triplicate. E_max_ and EC_50_ values are shown in Table 2.

**Figure 4:**
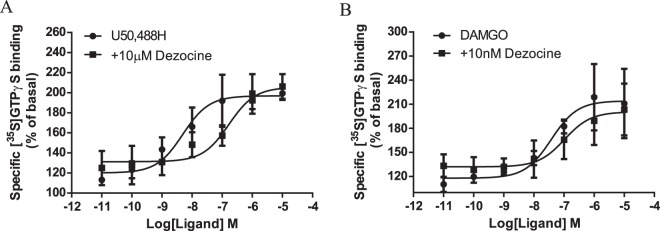
Antagonism of U50,488 H and DAMGO-induced [^35^S]GTPγS binding by Dezocine. Dezocine, at a concentration of 10 nM or 10 μM, was co-incubated with DAMGO or U50,488 H. Each data point represents the mean ± SEM of at least three independent experiments conducted in triplicate. *Ke* values are shown in Table 2.

To characterize the efficacy and potency of Dezocine, the [^35^S]GTPγS binding assay was conducted. As shown in Fig. 3A–C and Table 2, DAMGO and U50,488 H, as reference compounds, exhibited full agonist efficacy for G protein activation by μ and κ opioid receptors, but Dezocine-stimulated [^35^S]GTPγS was 33.6% and 45.8% of the maximal stimulation obtained with U50,488 H and DAMGO, respectively. As shown in Fig. 4 and Table 3, when co-incubated with U50,488 H, Dezocine caused a rightward shift in the concentration-response curves with a *Ke* value of 324.1 nM. When co-incubated with DAMGO, Dezocine also caused a rightward shift with a *Ke* value of 10.4 nM. These results suggested that Dezocine had more potent antagonist action against μ agonist than that against κ agonist. All together, the data obtained from *in vitro* functional assay indicated Dezocine acted as a κ partial agonist and μ partial agonist.

**Table 2:** Functional activity of Dezocine at human κ-, rat μ, or rat δ-opioid receptors.

Compounds	Agonist activity						Antagonist activity		
	MOR		KOR		DOR		MOR	KOR	DOR
	EC_50_ (nM)	E_max_ (%)	EC_50_ (nM)	E_max_ (%)	EC_50_ (nM)	E_max_ (%)	*Ke* (nM)	*Ke* (nM)	*Ke* (nM)
DAMGO	16.1 ± 0.3	240.7 ± 33.8	c	c	c	c	c	c	c
U50,488 H	c	c	5.6 ± 1.2	198.9 ± 7.5	c	c	c	c	c
DPDPE	c	c	c	c	1.4 ± 0.2	231.3 ± 7.1	c	c	c
Dezocine	b	45.8 ± 1.1^d^	b	33.6 ± 2.4^d^	b	26.1 ± 1.0^d^	10.4 ± 4.9	324.1 ± 23.6	a

Membranes were incubated with varying concentrations of ligands in the presence of 0.1 nM [^35^S]GTPγS. Data are expressed as the mean ± SEM for at least three independent experiments performed in triplicate.

^a^Not tested because of low affinity at DOR. ^b^Low stimulation at 10 μM. ^c^Not applicable. ^d^The agonist efficacy at 10 μM.

**Table 3:** ED_50_ values of the antinociception produced by Dezocine and Morphine.

Compounds	Antinociception ED_50_ (mg/kg)			
	Hot plate test	Abdominal constriction test	Formalin test Phase I Phase II	
Dezocine	—	0.2 (0.1–0.2)	0.4 (0.2–1.0)	0.4 (0.2–0.9)
Morphine	6.9 (5.6–8.5)^a^	0.8 (0.5–1.2)^a^	5.1 (4.3–5.9)^b^	3.1 (2.8–3.3)^b^

The antinociceptive ED_50_ values were calculated from data obtained at 15 min after drug administration. In parentheses are 95% confidence limits.

^a,b^The data of morphine associated with hot-plate test, abdominal constriction test and formalin test were cited from our previous data^34,35^.

### Antinociceptive effects of Dezocine

**Figure 5:**
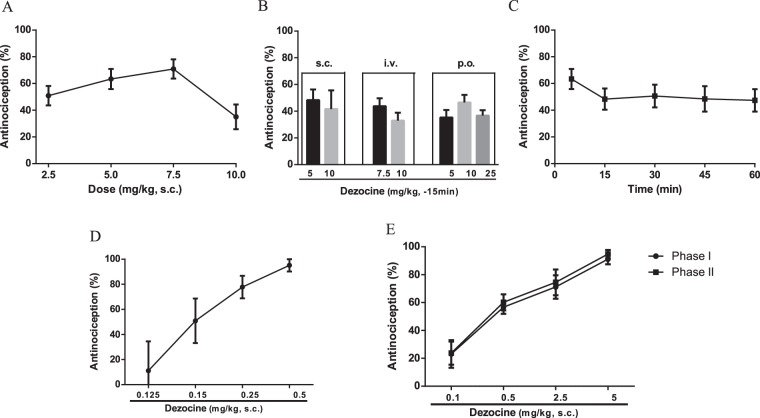
The antinociceptive effects of Dezocine evaluated by the hot plate test (**A**), abdominal constriction test (**D**) and formalin test (**E**). (**B**) The analgesic effects of Dezocine following different routes of administration. (**C**) Time courses for the effects of Dezocine in producing antinociception. After injected with various doses of Dezocine and the reference drug morphine, the antinociception was measured. ED_50_ values are shown in Table 3. Data are presented as mean ± SEM from at least 10 mice.

The antinociceptive property of Dezocine was characterized in different acute pain models. In the hot-plate test, a bell-shaped dose-response curve was obtained with Dezocine (Fig. 5A). The maximal antinociceptive effect was around 60% at 7.5 mg/kg, and higher dose (10 mg/kg) was not able to further increase the antinociception. Thus, the estimated antinociceptive ED_50_ of Dezocine in the hot-plate test was not obtained. To improve thermal antinociceptive efficacy, different administration routes, i.v., s.c., or p.o. were carried out. However, whatever the route of administration, Dezocine produced maximal antinociception around 55% (Fig. 5B). A fast onset of action was observed at 5 min, and the consistent high (close to peak) antinociceptive response up to 1 h was observed (Fig. 5C).

In the abdominal constriction test, Dezocine produced a full-dose response curve with an ED_50_ value of 0.2 mg/kg (Fig. 5D, Table 3), which was 4-fold more potent than morphine (ED_50_: 0.8 mg/kg, Table 3). In the formalin test, Dezocine also produced a full dose-response curve in both phase I and phase II, with ED_50_ values of 0.4 mg/kg (Fig. 5E, Table 3), which were approximately 13-fold more potent than morphine (ED_50_: 5.1 mg/kg) in the phase I and 9-fold more potent than morphine in the phase II (ED_50_: 3.1 mg/kg).

### Agonist properties of Dezocine

**Figure 6:**
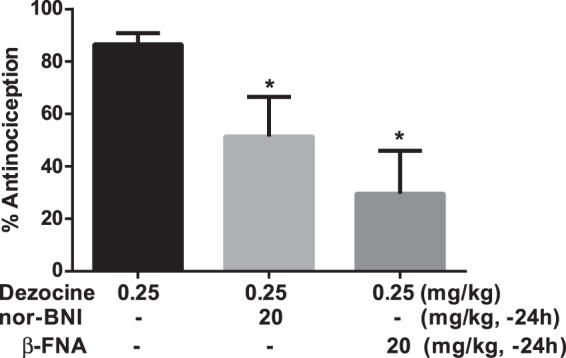
The Antinociceptive effects of Dezocine were mediated by κ and μ receptors in the abdominal constriction test. Mice were pretreated with 20 mg/kg nor-BNI and β-FNA for 24 h, then injected with 0.25 mg/kg Dezocine. Acetic acid solution was injected 15 min after drug administration. Data are presented as mean ± SEM from at least 10 mice. *p < 0.05 in comparison with Dezocine alone.

Next, the agonist effect produced by Dezocine in the abdominal constriction test was determined by the use of selective antagonists. As shown in Fig. 6, one-way ANOVA assay revealed that the treatments induced significant changes [F(2,27) = 4.867, p < 0.05]. The following Tukey’s comparison test showed that κ antagonist nor-BNI (p < 0.05) and μ antagonist β-FNA (p < 0.05) significantly decreased Dezocine-produced antinociception, indicating that the antinociceptive action of Dezocine was mediated by both the κ and μ opioid receptors.

### Antagonist properties of Dezocine

**Figure 7:**
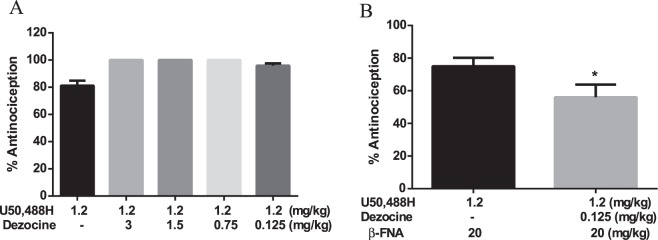
Effects of Dezocine on κ agonist U50,488H-induced antinociception. (**A**) Dezocine did not affect U50,488H-produced antinociceptive effects. (**B**) In the presence of μ antagonist β-FNA, pretreatment with Dezocine significantly decreased the antinociceptive effects produced by U50,488 H. Mice were injected with U50,488 H and Dezocine with or without β-FNA. After 15 min, the antinociception was measured in the abdominal constriction test. Data are presented as mean ± SEM from at least 10 mice. *p < 0.05 in comparison with U50,488 H + β-FNA group.

**Figure 8:**
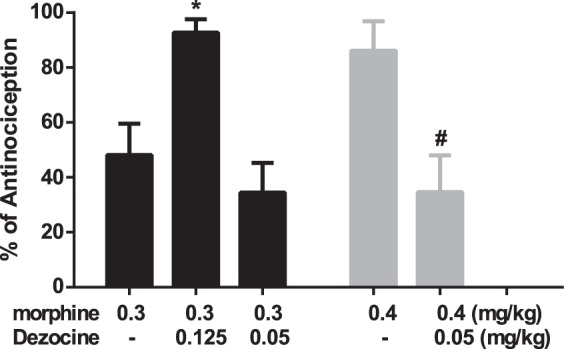
Effects of Dezocine on μ agonist morphine-induced antinociception. Mice were injected with morphine with or without Dezocine. After 15 min, the antinociception was measured in the abdominal constriction test. Data are presented as mean ± SEM from at least 10 mice. *p < 0.05 in comparison with 0.3 mg/kg morphine, ^#^*p* < 0.05 in comparison with 0.4 mg/kg morphine.

Our *in vitro* data indicated that Dezocine exhibited partial agonist activity at μ and κ opioid receptors, suggesting that Dezocine could act as a κ and μ antagonist, in addition to its agonist activity. To assess the antagonist activity of Dezocine, Dezocine and κ agonist U50,488 H or μ agonist morphine were administered concomitantly. As shown in Fig. 7A, Dezocine pretreatment did not inhibit U50,488H-induced antinociception. However, in the presence of μ antagonist β-FNA, Dezocine significantly decreased the antinociceptive effects produced by U50,488 H (p < 0.05, Fig. 7B). We further found Dezocine, at the dose of 0.05 mg/kg, significantly decreased the antinociceptive effects produced by 0.4 mg/kg morphine (p < 0.05), but did not affect the action produced by 0.3 mg/kg morphine (Fig. 8). Co-administration of 0.125 mg/kg Dezocine and 0.3 mg/kg morphine produced higher antinociception than morphine used alone (p > 0.05, Fig. 8).

### Sedative side effect of Dezocine

**Figure 9:**
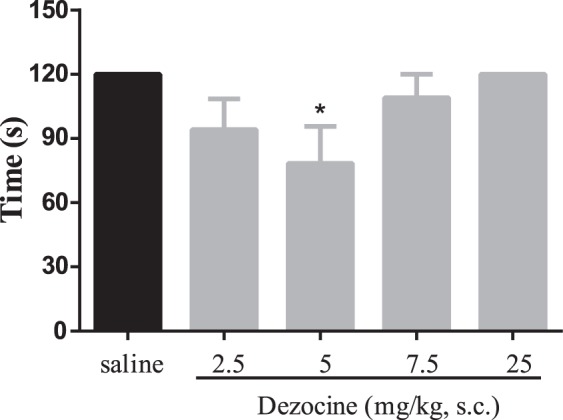
The sedative effects of Dezocine in rotorod test. Mice were injected with various doses of Dezocine, after 15 min, the mice were put on the rotorod apparatus, the time duration for each mice to fall off was recorded. Data are presented as mean ± SEM from at least 10 mice. *p < 0.05 in comparison with saline.

Activation of κ opioid receptors causes severe side effects of sedation, which is undesirable for therapeutic use. To study the sedative effects of Dezocine, the mice ability to maintain their position on an accelerating rotarod was detected. As shown in Fig. 9, animals were injected with various doses of Dezocine (2.5–25 mg/kg), then the rotarod test was conducted. Dezocine, at a dose of 5 mg/kg, produced significant sedation (p < 0.05), when the dose increased (7.5 and 25 mg/kg), the sedative effect diminished. There was a trend that 2.5 mg/kg Dezocine caused sedation, but it did not reach significant difference (p = 0.0881).

## Discussion

Our *in vitro* binding studies indicated that Dezocine produced high affinity at μ opioid receptor with Ki value of 1.46 nM, which was 15- and 273-fold selectivity for μ over κ and δ opioid receptors. These binding affinity results of Dezocine were consistent with the published data^17,19^. By using functional [^35^S]GTPγS binding assay, we demonstrated that Dezocine acted as a μ partial agonist, which was also consistent with the well-known low activity of Dezocine to stimulate μ receptors^8^. We further demonstrated that Dezocine was a κ partial agonist, which was different from the existing literatures showing that Dezocine acted as a κ antagonist^17,18^. We found that Dezocine produced maximal stimulation of [^35^S]GTPγS binding up to 33–45% mediated by κ receptors. Meanwhile, Dezocine also exhibited inhibitory effect on κ agonist U50,488H-stimulated [^35^S]GTPγS binding. Thus, Dezocine was termed by us as a κ partial agonist because, on its own, it had a lower intrinsic activity than full κ agonist U50,488 H, but in the presence of U50,488 H, it acted as a weak antagonist by preventing access to the κ receptor. Existing literature indicated that the functional action of Aripiprazole, a partial agonist at Dopamine D2 receptors, might vary among agonist, partial agonist or antagonist depending upon the level of receptor expression, function examined, and assessment assay applied^20,21^.

Considering that *in vitro* assay may not accurately reflect the action of Dezocine *in vivo*, the behavioral experiments in mice were conducted. In the abdominal constriction and formalin tests, we found that Dezocine produced full dose-response curves and displayed more potent antinociceptive response than morphine. In agreement, Dezocine was proved substantially more potent than morphine and pentazocine in the rodents and monkey^4^. In contrast to this sigmoid curve, in the hot plate test (55 °C), Dezocine-induced antinociception exhibited a bell-shaped dose-response curve. The similar bell-shaped dose-response curve was displayed in the antinociceptive action of pentazocine and buprenprphine^22–26^. Buprenorphin acted as κ antagonist^27^ and μ partial agonist^23,24,28^ and pentazocine acts as a κ agonist and μ partial agonist^29^. Thus, it seems that the plausible explanation for the observed ceiling effect on antinociception is that these compounds behave as μ partial agonists. The exact rationale for a ceiling effect of Dezocine-produced antinociception in the hot-plate test was unclear. but the possibility was suggested by data developed by the Lutfy group^25^, showing that the bell-shaped dose-response curve of buprenorphine was eliminated by the ORL-1 receptor antagonist and speculated that activation of ORL-1 receptors compromised the receptor-mediated antinociceptive action of buprenorphine. Shu *et al*. (2011) found that nor-BNI enhanced the antinociception by pentazocine and turned the later descending portion of the bell-shaped dose-response curve into a sigmoid curve and proposed that activation of κ receptor compromised the μ receptor-mediated antinociception of pentazocine^26^. Thus, it is possible that Dezocine activates other molecular targets, and compromises opioid receptor-mediated actions of Dezocine. Indeed, in addition to opioid receptors, Dezocine also targets amine transporter proteins^17^.

We further found that the antinociceptive action of Dezocine was inhibited by κ antagonist nor-BNI and μ antagonist β-FNA, supporting our *in vitro* conclusion that Dezocine had κ and μ agonist activity. It is believed that Dezocine’s clinical efficacy and little chance to provoke adverse events during the therapeutic process are mainly attributed to its partial agonist activity at the μ opioid receptors^30^. However, we demonstrated that the antinociceptive response of Dezocine was a result of action at both the κ and μ opioid receptors. Liu *et al*. (2014) demonstrated that in addition to opioid receptors, Dezocine also targeted norepinephrine and serotonin reuptake^17^. Wang *et al*. (2017) further found that Dezocine produced antihypersensitivity activity in neuropathic pain through its μ activation and norepinephrine reuptake inhibition (NRI)^31^. The activity of Dezocine at non-opioid sites in acute pain modulation was not investigated in the present work.

To assess the antagonist activity of Dezocine at the κ receptor *in vivo*, the animals were pretreated with Dezocine before giving a κ agonist U50,488 H. We noticed that Dezocine did not inhibit the antinociceptive effect of U50,488 H. A possible explanation for this finding was that Dezocine-suppressed U50,488 H action was compromised by its own μ agonist activity. Indeed, in the presence of μ antagonist β-FNA, Dezocine exhibited κ antagonist activity as demonstrated by its ability to decrease U50,488H-induced antinociception. On the other hand, we found that 0.05 mg/kg Dezocine significantly inhibited μ agonist morphine-induced antinociception, indicating its μ antagonist activity. Whereas co-administration of 0.125 mg/kg Dezocine with morphine produced higher antinociception than morphine used alone. This phenomenon can also be rationalized as resulting from Dezocine-induced κ receptor activation. Thus, our results may explain the controversial findings within the literature concerning the benefit of Dezocine and other opioid analgesic drugs in combination. Dezocine, as a μ partial agonist, was able to antagonize morphine analgesia^32^, but due to its κ agonist activity, combination of Dezocine and morphine might produce higher analgesic effects than morphine alone^15,33^. The action of Dezocine may vary depend on the dosage used. The accurate interactions between Dezocine and morphine or other opioids need further investigation.

The partial agonist activity is generally believed to support it safety, such as buprenorphine displays a ceiling effect on μ receptor-mediated respiratory depression^4,16^. Hence, we assume that Dezocine can produce lower κ receptor activation-associated sedative side effects. Indeed, in the present work, we found that Dezocine exhibited limited sedative effects with a ceiling effecting at 5 mg/kg. Moreover, attributed to the fact that the ED_50_ values of Dezocine-induced antinociception was around 0.2–0.4 mg/kg, the dose of Dezocine that produced sedative side effects (5 mg/kg) was much higher than those to produce antinociception. Thus, the partial agonist activity of Dezocine supports it unique property for clinical application with less side effects.

Taken together, Dezocine was demonstrated as a κ partial agonist and μ partial agonist. In mice model of acute pain, Dezocine-produced antinociception was mediated by both the κ and μ receptor activation. Dezocine displayed limited sedative effects with a ceiling effecting at a moderate dose. Combined with other opioid drugs, the action of Dezocine may vary depend on the dosage used.

## Methods

### Drugs

(±)-trans-U-50488H (U50,488 H), [D-Ala2,N-Me-Phe4,Gly5-ol]-enkephalin (DAMGO) [D-Pen2,5,P-CL-Phe4]Enkephalin (DPDPE), nor-binaltorphimine (nor-BNI), β-funaltrexamine (β-FNA), naloxone, GTPγS and GDP were obtained from Sigma-Aldrich (St. Louis, MO). [^3^H]DAMGO (51.5 Ci/mmol), [^3^H]DPDPE (57.4 Ci/mmol), [^3^H]U69593 (43.6 Ci/mmol) and [^35^S]GTPγS (1250 Ci/mmol) were bought from Perkin Elmer.

### *In vitro* studies

#### Cell culture

Chinese hamster ovary (CHO) cells stably expressing human κ-, rat μ, or rat δ-opioid receptors were maintained in F12 medium (Gibco) with 10% fetal serum and 0.25 mg/ml (G418) (Roche). Cells were incubated in a humidified atmosphere consisting 95% air and 5% CO_2_ at 37 °C, and were seeded in 10 cm dishes. Cells were collected when reached 85% confluence, and the cell membrane was prepared.

#### Cell membrane preparation

Cells were washed with phosphate buffer saline (PBS), detached by incubation with PBS containing 1 mM EDTA, and centrifuged at 1000 g for 10 min. The cell pellet was suspended and homogenized in ice-cold homogenization buffer (50 mM HEPES, 1 mM MgCl_2_, 1 mM EGTA, pH 7.4,). After centrifugation at 40,000 g for 10 min (4 °C), pellets were resuspended, homogenized and centrifuged again. The final pellets were resuspended in Tris–HCl buffer (50 mM, pH 7.4). Protein concentration was detected and the aliquots were stored at −80 °C.

#### Receptor binding assay

Ligand binding assay was carried out according to our work^34,35^. Competition inhibition of [^3^H]ligands binding opioid receptors by Dezocine, U50,488 H, DAMGO or DPDPE was performed in the absence or presence at various concentration of each compound. The binding assay was performed in Tris–HCl buffer (50 mM, pH 7.4) at 37 °C for 30 min in triple in a final volume of 0.5 ml containing 30 μg of membrane protein and [^3^H]ligands ([^3^H]U69593 1.5 nM; [^3^H]DAMGO 2.0 nM; [^3^H]DPDPE 1.1 nM). Naloxone (10 μM) was used to define nonspecific binding. Bound and free [^3^H]ligands were separated by filtration under reduced pressure with GF/B filters (Whatman). Radioactivity was determined by liquid scintillation counter (Beckman LS6500).

#### [^35^S]GTPγS binding assay

[^35^S]GTPγS binding assay was carried out according to our work^34,35^. Briefly, membranes (15 μg) were incubated with 0.1 nM [^35^S]GTPγS in reaction buffer (50 mM Tris-HCl, 1 mM EDTA, 5 mM MgCl_2_, 100 mM NaCl, PH7.4) and 40 μM GDP at 30 °C for 1 h in the presence of increasing concentrations of Dezocine, U50,488 H, DAMGO or DPDPE. Nonradioactive GTPγS (10 μM) was used to define nonspecific binding. Radioactivity was determined by liquid scintillation counter (Beckman LS6500). The percentage of stimulated [^35^S]GTPγS binding was calculated as 100× (cpm sample−cpm nonspecific)/(cpm basal−cpm nonspecific). The potency of an antagonist is defined by its *Ke* (equilibrium dissociation constant) value, as determined by the “single” dose method. The agonists (U50, 488H and DAMGO) dose-response curves were generated in the absence and presence of Dezocine. The *Ke* value is calculated according to the equation:

$$[{\rm{test}}\,{\rm{drug}}]/({{\rm{EC}}}_{50-2}/{{\rm{EC}}}_{50-1}-1),$$

where EC_50-2_ value is the EC_50_ value in the presence of the test drug and EC_50−1_ is the value in the absence of the test drug^36–38^.

### *In vivo* studies

#### Animals

Kunming strain male and female mice weighing 20 to 24 g were obtained from the Laboratory Animal Center, Chinese Academy of Sciences (Shanghai, China). Mice were housed in groups in a room maintained in a 12/12 h light/dark cycle in a temperature controlled environment with free access to food and water. All research protocols were approved by the Animal Care and Use Committee of Shanghai Institute of Materia Medica, Chinese Academy of Sciences. All experimental procedures were in strict accordance with the National Institutes of Health Guide for the Care and Use of Laboratory Animals.

### Antinociceptive tests

#### Hot plate test

Hot plate test was performed according to the method described previously^31,34,35^. Briefly, mice were placed on a 55 °C heated surface, the latency to licking or jumping was recorded. The cut-off time of 60 s was used to minimize tissue damage. Before formal study, the nociceptive response of each mouse was measured three times, and the mean of the 2^nd^ and 3^rd^ responses was used as pre-drug latency. The mouse not responding within 20 s was excluded. The percentage antinociception was calculated as 100× [(test latency-predrug latency)/(cut-off time -predrug latency)].

#### Abdominal constriction test

The abdominal constriction test was conducted according to our previous work^31,35^. An abdominal constriction was defined as a wave of contraction of the abdominal musculature followed by extension of the hind limbs after injected with 0.6% of acetic acid (10 ml/kg body weight, i.p.). The number of abdominal constriction was counted for 15 min after acetic acid administration. Percentage of analgesia was expressed as 100× (No. of mean control abdominal constriction−No. of test abdominal constriction)/No. of mean control abdominal constriction.

#### Formalin test

The formalin test was performed according to our previous work^35^. In brief, the amount of time that mice spent licking or flinching the injected hind paw was recorded after injected with 20 μl 1.0% formalin at the plantar surface of the right hind paw. The mice were observed fro 60 min. The first 10 min was recorded as the phase I, and the rest of time was recorded as phase II. Percentage of analgesia was calculated as 100× (time of control – time of drug)/time of control.

#### Agonist effect of Dezocine

To define the agonist profile of Dezocine, mice were pretreated with selective κ antagonist nor-BNI (20 mg/kg, i.p., −24 h), μ antagonist β-FNA (20 mg/kg, i.p., −24 h) or saline^31,34,35^, then mice were administrated with Dezocine. After 15 min, the antinociceptive effect was assessed.

#### Antagonist effect of Dezocine

To define the κ antagonist profile of Dezocine, mice were pretreated with various doses of Dezocine or saline. 5 min later, the mice were administrated with κ agonist U50,488 H (1.2 mg/kg, i.p). After 15 min, the antinociceptive effect was assessed. To avoid Dezocine’s μ agonist activity contributing to its antinociception, mice were pretreated with μ antagonist β-FNA before Dezocine and U50,488 H co-administration.

The μ antagonist effect of Dezocine was also determined by pretreatment with Dezocine followed by μ agonist morphine injection. After 15 min, the antinociceptive effect was assessed.

#### Rotorod test

The test was conducted in mice according to the method described previously with slight modification^31^. In brief, mouse was individually trained to maintain their position on a rotorod for 120 s or more, and the mice falling off within 120 s were excluded. 15 min after Dezocine injection, mice were put on the rotorod apparatus, the time duration for each mouse to fall off was recorded.

#### Statistical Analysis

Curve-fitting analysis was performed using GraphPad Prism 6.0 software. Data represents the mean ± SEM of at least three separate experiments. Statistical significance was determined by one-way ANOVA followed by post hoc comparison using Tukey’s tests. When only two groups were compared, statistical significance was determined by unpaired student’s *t* test.

## Data Availability

All data generated or analyzed during this study are included in this published article.
